# Inotersen preserves or improves quality of life in hereditary transthyretin amyloidosis

**DOI:** 10.1007/s00415-019-09671-9

**Published:** 2019-12-18

**Authors:** Teresa Coelho, Aaron Yarlas, Marcia Waddington-Cruz, Michelle K. White, Asia Sikora Kessler, Andrew Lovley, Michael Pollock, Spencer Guthrie, Elizabeth J. Ackermann, Steven G. Hughes, Chafic Karam, Sami Khella, Morie Gertz, Giampaolo Merlini, Laura Obici, Hartmut H. Schmidt, Michael Polydefkis, P. James B. Dyck, Thomas H. Brannagan III, Isabel Conceição, Merrill D. Benson, John L. Berk

**Affiliations:** 1 Hospital Santo António, Centro Hospitalar Universitário do Porto, Porto, Portugal; 2 Optum, 1301 Atwood Avenue, Johnston, RI 02919 USA; 3 Hospital Universitário Clementino Fraga Filho, Universidade Federal do Rio de Janeiro, Rio de Janeiro, Brazil; 4 Akcea Therapeutics, Boston, MA USA; 5 Aurora Bio, San Francisco, CA USA; 6 Otonomy, Inc., San Diego, CA USA; 7 Organovo Holdings, Inc., San Diego, CA USA; 8 Department of Neurology, Oregon Health and Science University, Portland, OR USA; 9 University of Pennsylvania, Philadelphia, PA USA; 10 Division of Hematology, Mayo Clinic, Rochester, MN USA; 11 Amyloidosis Research and Treatment Center, Fondazione IRCCS Policlinico San Matteo, University of Pavia, Pavia, Italy; 12 University of Münster, Münster, Germany; 13 Johns Hopkins University School of Medicine, Baltimore, MD USA; 14 Department of Neurology, Mayo Clinic, Rochester, MN USA; 15 Columbia University, New York, NY USA; 16 Faculdade de Medicina, Hospital de Santa Maria, CHULN, Universidade de Lisboa, Lisboa, Portugal; 17 Indiana University School of Medicine, Indianapolis, IN USA; 18 Boston University School of Medicine, Boston, MA USA

**Keywords:** Transthyretin amyloidosis, Quality of life, Polyneuropathy, Physical function, Rare disease

## Abstract

**Objective:**

To examine the impact on quality of life (QOL) of patients with hATTR amyloidosis with polyneuropathy treated with inotersen (Tegsedi™) versus placebo.

**Methods:**

Data were from the NEURO-TTR trial (ClinicalTrials.gov Identifier: NCT01737398), a phase 3, multinational, randomized, double-blind, placebo-controlled study of inotersen in patients with hATTR amyloidosis with polyneuropathy. At baseline and week 66, QOL measures—the Norfolk-QOL-Diabetic Neuropathy (DN) questionnaire and SF-36v2^®^ Health Survey (SF-36v2)—were assessed. Treatment differences in mean changes in QOL from baseline to week 66 were tested using mixed-effect models with repeated measures. Responder analyses compared the percentages of patients whose QOL meaningfully improved or worsened from baseline to week 66 in inotersen and placebo arms. Descriptive analysis of item responses examined treatment differences in specific activities and functions at week 66.

**Results:**

Statistically significant mean differences between treatment arms were observed for three of five Norfolk-QOL-DN domains and five of eight SF-36v2 domains, with better outcomes for inotersen than placebo in physical functioning, activities of daily living, neuropathic symptoms, pain, role limitations due to health problems, and social functioning. A larger percentage of patients in the inotersen arm than the placebo arm showed preservation or improvement in Norfolk-QOL-DN and SF-36v2 scores from baseline to week 66. Responses at week 66 showed more substantial problems with daily activities and functioning for patients in the placebo arm than in the inotersen arm.

**Conclusion:**

Patients with hATTR amyloidosis with polyneuropathy treated with inotersen showed preserved or improved QOL at 66 weeks compared to those who received placebo.

## Introduction

Hereditary transthyretin (hATTR) amyloidosis is a rare, systemic, progressive, debilitating, and fatal disease characterized by mutations in the gene encoding the transthyretin (TTR) protein [1]. More than 130 reported TTR gene mutations promote misfolding of TTR proteins, which aggregate and deposit as insoluble amyloid deposits in tissues, inducing organ damage [2]. Worldwide prevalence estimates indicate that approximately 50,000 people have been diagnosed with hATTR amyloidosis, although it is thought to be significantly underdiagnosed [3]. Patients with hATTR amyloidosis typically suffer peripheral and autonomic nerve fiber injury, resulting in a length-dependent sensorimotor peripheral neuropathy, manifesting as pain, numbness, and weakness with eventual loss of ambulation. Others experience cardiomyopathy, characterized by conduction disease, thickening and stiffening of the ventricles, and eventual heart failure [1, 4–6]. The majority of patients, however, exhibit a mixed phenotype including nerve- and heart-related manifestations [7].

Patients with hATTR amyloidosis experience severely compromised quality of life (QOL) [8–11] that worsens with disease progression [12]. Furthermore, patients with hATTR amyloidosis with polyneuropathy have significantly impaired physical functioning and well-being [9, 10]. Neuropathy-related QOL for patients with hATTR amyloidosis with polyneuropathy is comparable to patients with severe diabetic neuropathy that resulted in ulceration, gangrene, or amputations [11].

The NEURO-TTR trial was a phase 3 clinical trial that examined the safety and efficacy of inotersen (Tegsedi™) in patients with hATTR amyloidosis with polyneuropathy [13]. About two thirds of patients also had cardiomyopathy. The current research examines the impact of 66 weeks of inotersen treatment on patients’ QOL recorded in the NEURO-TTR trial.

## Methods

### Sample and study characteristics

The NEURO-TTR trial (ClinicalTrials.gov Identifier: NCT01737398) was a phase 3, multinational, randomized, double-blind, placebo-controlled study of inotersen in patients with hATTR amyloidosis with polyneuropathy. Patients were randomized to receive 300 mg inotersen or placebo in a 2:1 ratio within each of three strata: presence/absence of previous treatment with tafamidis and/or diflunisal; Stage 1 (ambulatory without assistance) or Stage 2 (ambulatory with assistance) neurologic disease severity, according to Coutinho et al.’s staging [14]; and Val30Met mutation or non-Val30Met mutation. Study drug was administered subcutaneously on three alternating days during the first week and once per week for the following 65 weeks.

Patients were eligible for the study if they were at least 18 years old, had Stage 1 or Stage 2 disease severity, had a neuropathy impairment score (NIS) between 10 and 130 (inclusive) at screening, had a documented TTR variant by genotyping; and had documented amyloid deposits by biopsy. Exclusion criteria included previous or anticipated liver transplant within 1 year of screening, a Karnofsky performance status ≤ 50, a New York Heart Association (NYHA) functional classification ≥ 3, or presence of type 1 or 2 diabetes, human immunodeficiency virus (HIV), hepatitis B, or hepatitis C.

Primary endpoints of the trial were changes from baseline to week 66 in the modified Neuropathy Impairment Score + 7 (mNIS + 7) [15–17] composite score and in the total score of the Norfolk QOL–Diabetic Neuropathy (DN) questionnaire [18], which has been validated in patients with ATTR accompanied by polyneuropathy [19]. A tertiary endpoint of the trial was change from baseline to week 66 in the physical component summary (PCS) score of the SF-36v2. Inotersen was shown to be significantly efficacious relative to placebo for each of these outcomes [13]. Safety outcomes for the NEURO-TTR trial have also been published [13].

The Norfolk QOL-DN and the SF-36v2 were administered to patients at baseline, week 35, and week 66 (end of treatment). All analyses were based on the full analysis set, which was comprised of all randomized patients who received at least one injection of study drug and who had at least one post-baseline efficacy assessment for the mNIS + 7 score or Norfolk QOL-DN questionnaire total score.

### Outcomes

Neuropathic-specific QOL was measured using the 35-item Norfolk QOL-DN [18]. It yields a total score based on all 35 items (score range from − 4 to 136), and scores on five subscales capturing outcomes associated with damage to nerve fibers: activities of daily living (ADLs; five items, score range 0–20); autonomic neuropathy (three items, score range 0–12), large fiber neuropathy/physical functioning (15 items, score range − 4–56); small fiber neuropathy (four items, score range 0–16); and symptoms (eight items, score range 0–32). In all cases, higher scores indicate worse functioning.

Generic QOL was assessed using the SF-36v2 (with 4-week recall), a 36-item patient-reported outcome measure of functional health and well-being [20]. Responses to SF-36v2 items can be combined to compute scores for eight domains of QOL: physical functioning, role limitations due to physical health (role-physical), bodily pain, perception of general health, vitality, social functioning, role limitations due to emotional health (role-emotional), and mental health. Two summary scores—PCS, capturing global physical health, and the mental component summary (MCS) capturing global mental health—are calculated using weighted combinations of scores from all eight domains. All SF-36v2 domains and summary scores are expressed as *T* scores using norm-based methods, standardized to a mean of 50 and a standard deviation of 10 in the general population. Higher SF-36v2 scores reflect better QOL. The SF-36v2 has been previously used in randomized-controlled trials comparing disease progression and QoL in patients with hATTR polyneuropathy [21].

Responder-level minimally important change (MIC) values have been established for SF-36v2 domain and summary scores using distribution-based methods. MIC threshold values, which have been defined as the smallest differences in scores that an individual patient would consider as beneficial and for which a clinician would recommend adjusting patients’ care [22], can facilitate interpretation of whether a patient’s change in health is clinically meaningful.

### Statistical analysis

All analyses described here were exploratory and post hoc.

Mixed-effects models for repeated measures (MMRM) were used to test for treatment arm differences in mean change-from-baseline scores for each domain of the Norfolk QOL-DN and SF-36v2 at week 66. Fixed class effects for all models included treatment arm, visit (week 35 and week 66), each of the three stratification factors—previous treatment with tafamidis and/or diflunisal (presence vs. absence), disease severity (Stage 1 vs. Stage 2), and mutation type (Val30Met mutation vs. non-Val30Met mutation)—and the treatment arm * visit interaction. Covariates included baseline score and the baseline score * visit interaction. Degrees of freedom were estimated using the Kenward–Rogers approach. An unstructured covariance model was used for within-subject residuals. Pairwise comparisons tested for treatment arm differences in least-squares (LS) mean change scores at week 66.

Responder analysis examined the proportion of patients in each treatment arm who, at week 66, showed worse, same, or better scores from baseline for each SF-36v2 and Norfolk QOL-DN domain and summary measure. For SF-36v2 domain and summary scores, change status was determined based on established responder-level MICs. Patients whose SF-36v2 score at week 66 fell below their score at baseline by a magnitude of at least the MIC for that outcome were categorized as “worse,” while those whose score at week 66 fell below their score at week baseline by less than the MIC for that outcome were categorized as “same/better.” Patients whose SF-36v2 score at week 66 exceeded their score at baseline by a magnitude of at least the MIC for that outcome were categorized as “better.”

For the Norfolk QOL-DN domains and total score, which do not have established responder-level MICs, change status was determined based on the distribution for each domain and total score. Specifically, status as “better,” “same,” or “worse” was determined using 0.5 of a standard deviation (SD) of the baseline score, as this magnitude has been identified as being indicative of an MIC in a score [23, 24]. Patients whose Norfolk QOL-DN score at week 66 met or exceeded their score at baseline by a magnitude of at least 0.5 of an SD for that outcome were categorized as “worse,” while those who did not exceed their score at baseline by a magnitude of at least 0.5 of an SD for that outcome were categorized as “same/better.” Patients whose Norfolk QOL-DN score at week 66 fell below their score at baseline by a magnitude of at least 0.5 of an SD for that outcome were categorized as “better.” For each SF-36v2 and Norfolk QOL-DN score, the percentage of patients whose scores were categorized as same/better or better at week 66 than at baseline was compared across treatment groups using Fisher’s exact tests.

To provide a more concrete interpretation of changes in QOL as a function of treatment, changes from baseline to week 66 in patients’ responses to specific items of the Norfolk QOL-DN and SF-36v2 were assessed. The items included in the analyses ask how patients’ physical and emotional health impacts patients’ daily lives. For the Norfolk QOL-DN, items examine difficulty with ADLs and physical functioning; for the SF-36v2, items examine impairments in daily activities related to physical functioning, role-physical, bodily pain, and social functioning. Response choices for the selected items were dichotomized as indicating substantial and not substantial impairment. All examined Norfolk QOL-DN items used response options of ‘not a problem’, ‘very mild problem’, ‘mild problem’, ‘moderate problem’, and ‘severe problem’, with the latter two options coded as indicating substantial problems. SF-36v2 physical functioning items use three response options, ‘not limited at all’, ‘limited a little’, and ‘limited a lot’; only the last of these options was coded as indicating substantial impairment. Response choices for SF-36v2 role-physical items include ‘all of the time’, ‘most of the time’, ‘some of the time’, ‘a little of the time’, and ‘none of the time’; substantial impairment was coded for the first two of these options. The SF-36v2 bodily pain item “How much did pain interfere with your normal work, including both work outside the home and housework?” has five possible responses: ‘extremely’, ‘quite a bit’, ‘moderately’, ‘a little bit’, and ‘not at all’. Patients who indicated one of the first two responses were considered to have a substantial level of impairment, while those who selected one of the last three responses were considered to not have a substantial level of impairment. Analogous response choices were dichotomized similarly for the SF-36v2 social functioning item “During the past 4 weeks, to what extent has your physical health or emotional problems interfered with your normal social activities with family, friends, neighbors, or groups?” The percentages of patients whose item-level response indicated a substantial level of impairment at baseline and week 66 were calculated for patients in both treatment arms. Descriptive comparisons across treatment arms in the proportion of patients with responses indicating substantial impairment at baseline and at week 66 were examined to provide a real-world context for assessing the impact of inotersen on everyday functioning.

## Results

Baseline characteristics of the analysis sample are presented in Table 1. Patients did not differ statistically by treatment arm for any of the listed characteristics. A majority of sample patients (62%) had cardiomyopathy. Approximately two-thirds of the sample (68%) had Stage 1 neurologic disease severity; the remainder were Stage 2.

**Table 1:** Baseline patient characteristics in the NEURO-TTR trial full analysis set (*n* = 165)

	Inotersen (*n* = 106)	Placebo (*n* = 59)	*p *value (treatment arm difference)*
Age, mean (SD)	59.6 (12.4)	59.4 (14.1)	0.937
Female, *N* (%)	31 (29.2)	18 (30.5)	0.861
Mutation type, *N* (%)			0.626
Val30Met mutation	54 (50.9)	33 (55.9)	
Non-Val30Met mutation	52 (49.1)	26 (44.1)	
Modified BMI, mean (SD)	1025.3 (222.7)	1053.7 (228.5)	0.438
Presence of cardiomyopathy, *N* (%)	70 (66.0)	32 (54.2)	0.181
Neuropathy stage, *N* (%)			0.605
Stage 1	71 (67.0)	42 (71.2)	
Stage 2	35 (33.0)	17 (28.8)	
Duration of neuropathic symptoms in years, mean (SD)	5.4 (4.5)	5.4 (4.4)	0.947
Prior treatment with tafamidis/diflunisal, *N* (%)	62 (58.5)	35 (59.3)	1.000
mNIS + 7 total score, mean (SD)	79.4 (37.5)	74.1 (39.0)	0.399
Norfolk QOL-DN, mean (SD)			
Activities of daily living	6.5 (5.9)	6.4 (5.7)	0.909
Autonomic neuropathy	2.2 (2.8)	1.8 (2.7)	0.411
Large fiber/physical functioning	24.1 (15.4)	24.4 (13.7)	0.891
Small fiber	5.1 (4.2)	5.2 (4.5)	0.829
Symptoms	10.6 (6.1)	10.7 (6.5)	0.974
Total score	48.6 (28.2)	48.6 (27.0)	0.994
SF-36v2, mean (SD)			
Physical functioning	34.6 (9.8)	36.7 (10.6)	0.190
Role-physical	37.2 (10.7)	38.2 (10.2)	0.575
Bodily pain	43.5 (9.8)	42.6 (10.4)	0.582
General health	40.8 (8.8)	43.1 (9.1)	0.112
Vitality	45.9 (10.0)	46.5 (11.1)	0.720
Social functioning	43.7 (10.6)	44.5 (10.7)	0.629
Role-emotional	45.6 (10.1)	45.7 (11.1)	0.981
Mental health	49.4 (9.0)	48.9 (10.2)	0.714
Physical component summary	35.5 (8.9)	37.2 (9.9)	0.267
Mental component summary	51.1 (9.2)	50.6 (10.7)	0.774

*p values (two-tailed) are based on independent-samples *t *tests for continuous variables and Fisher’s exact tests for categorical variables

**Fig. 1:**
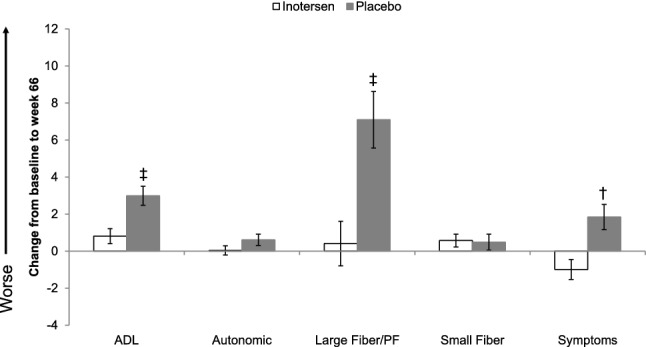
Change in mean Norfolk QOL-DN Domain Scores from baseline to week 66 by treatment arm. *ADL* activities of daily living, *PF* physical functioning. Error bars represent standard errors *p < 0.05, ^†^*p* < 0.01, ^‡^*p* < 0.001

Changes from baseline to week 66 in mean Norfolk QOL-DN domain scores by treatment arm are presented in Fig. 1. Statistically significant differences in scores between treatment arms were observed for three domains: ADLs, large fiber/physical functioning, and symptoms, where stabilization or improvements in the inotersen arm were contrasted with worsening in the placebo arm, all *p* ≤ 0.01.

**Fig. 2:**
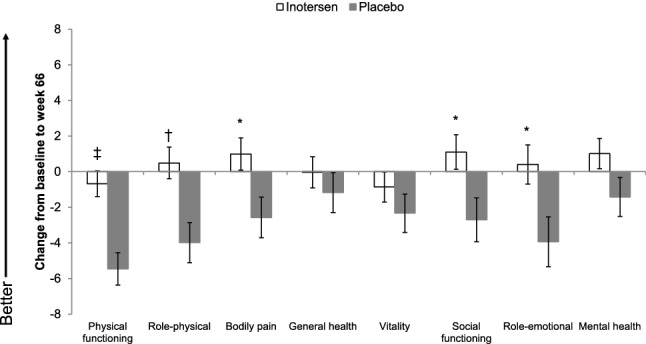
Change in mean SF-36v2 Domain Scores from baseline to week 66 by treatment arm. Error bars represent standard errors *p < 0.05, ^†^*p* < 0.01, ^‡^*p* < 0.001

Changes from baseline to week 66 in mean SF-36v2 domain scores by treatment arm are presented in Fig. 2. Statistically significant differences between treatment arms in change-from-baseline scores at week 66 were observed for five domains: physical functioning, role-physical, bodily pain, social functioning, and role-emotional, where stabilization or improvements in the inotersen arm were again contrasted with worsening in the placebo arm, all *p* < 0.05.

**Fig. 3:**
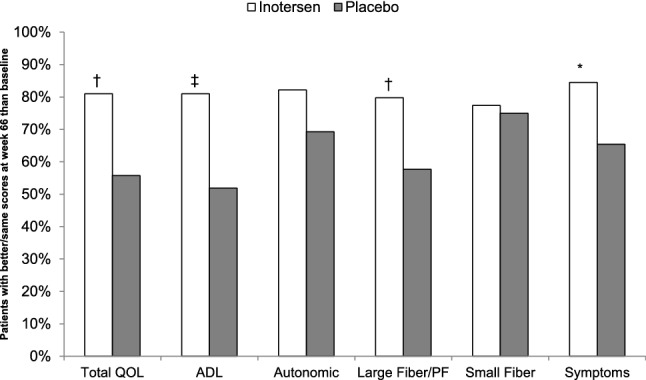
Responder analysis: percentage of patients with better or same Norfolk QOL-DN Scores at week 66 relative to baseline by treatment arm. *ADL* activities of daily living, *QOL* Quality of Life, *PF* physical functioning. Better/same scores defined as less than 0.5 of a standard deviation higher at week 66 than at baseline *p < 0.05, ^†^*p* < 0.01, ^‡^*p* < 0.001

Figure 3 presents the percentage of patients in each treatment arm whose Norfolk QOL-DN scores were the same or improved from baseline to week 66. Patients receiving inotersen had better/same Norfolk QOL-DN total scores at week 66 compared to baseline more often than did patients receiving placebo (81.0% vs. 55.8%, *p* = 0.0031). Statistically significantly higher percentages of patients with better/same scores at week 66 than baseline in the inotersen arm than in the placebo arm were also observed for the ADL domain (81.0% vs. 51.9%, *p* = 0.0005), large fiber/physical functioning domain (79.8% vs. 57.7%, *p* = 0.0068), and symptoms domain (84.5% vs. 65.4%, *p* = 0.0121). Patients receiving inotersen were more than twice as likely as those receiving placebo to have a meaningfully better Norfolk QOL-DN total score at week 66 than baseline (25.0% vs. 9.6%, *p* = 0.042). Statistically significantly higher percentages of patients with better scores at week 66 in the inotersen arm than in the placebo arm were also observed for the large fiber/physical functioning domain (27.4% vs. 7.7%, *p* = 0. 007).

**Fig. 4:**
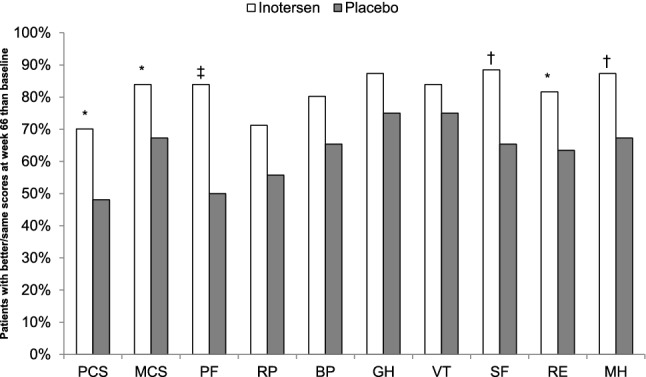
Responder analysis: percentage of patients with better/same SF-36v2 Scores at week 66 relative to baseline by treatment arm. *BP* bodily pain, *GH* general health, *MH* mental health, *MCS* mental component summary, *PCS* physical component summary, *PF* physical functioning, *RE* role-emotional, *RP* role-physical, *SF* social functioning, *VT* vitality. Better/same scores defined as greater than the minimally important change (MIC) threshold value lower at week 66 than at baseline *p < 0.05, ^†^*p* < 0.01, ^‡^*p* < 0.001

The percentages of patients whose SF-36v2 domain and summary scores were better or the same at week 66 than baseline are presented in Fig. 4. A significantly larger percentage of patients receiving inotersen than those receiving placebo showed better/same physical and mental summary scores at week 66: 70.1% vs. 48.1% (*p* = 0.0118) for PCS and 83.9% vs. 67.3% (*p* = 0.0343) for MCS. A statistically significantly larger percentage of inotersen patients than placebo patients showed better/same scores at week 66 on four domains: physical functioning (83.8% vs. 50.0%, *p* < 0.0001), social functioning (88.5% vs. 65.4%, *p* = 0.0018), role-emotional (81.6% vs. 63.5%, *p* = 0.0256), and mental health (87.4% vs. 67.3%, *p* = 0.0079). Differences in the role-physical (71.3% vs. 55.8%), bodily pain (80.2% vs. 65.4%), and general health (87.4% vs. 75.0%) domains were just outside statistical significance (all *p* = 0.07). Statistically significantly larger percentages of patients with better scores at week 66 in the inotersen arm than in the placebo arm were observed for the physical functioning domain (18.4% vs. 5.8%, *p* = 0.042) and the role-physical domain (39.1% vs. 17.3%, *p* = 0.008).

While the previously described findings were based on Norfolk QOL-DN and SF-36v2 scores at the domain level, additional analyses examined item-level responses for each of the two instruments. At week 66, responses to selected Norfolk QOL-DN items (Table 2) by patients receiving placebo more frequently indicated that they had a moderate or severe problem with a number of symptoms, daily activities, and physical functioning than patients receiving inotersen. For example, patients in the placebo arm indicated pain keeping them awake at night and being bothered by the touch of bedsheets more than twice as often as those receiving inotersen. Descriptively, a noticeably larger percentage of patients (i.e., at least 10% more) receiving placebo than inotersen reported problems with their symptoms affecting their usual activities; feeling unsteady on their feet; having difficulty moving fingers; having difficulty rising from a chair or descending stairs; having diarrhea; and having difficulty bathing, dressing, walking, getting on/off the toilet, and handling utensils.

**Table 2:** Percentage of patients with Norfolk QOL-DN item responses indicating substantial impairment at week 66, and change in percentage from baseline to week 66, by treatment arm

Item #	Content	Week 66		Change from baseline to week 66	
		Inotersen (%)	Placebo (%)	Inotersen (%)	Placebo (%)
8	Pain kept you awake at night	15.5	36.5	− 1.7	7.2
9	Touch of bed sheets bothered you	11.9	26.9	− 1.6	8.0
10	Injured yourself without feeling	14.3	19.2	1.0	5.4
11	Symptoms affect usual activity	35.7	50.0	6.9	20.7
12	Difficult movement with fingers	46.4	63.5	1.7	18.6
13	Felt unsteady on your feet	48.8	67.3	− 0.7	17.3
14	Problem getting out of a chair	50.0	61.5	3.3	11.5
15	Problem walking down stairs	41.7	57.7	0.7	16.3
16	Unable to feel your feet	45.2	40.4	10.3	0.7
17	Unable to tell hot (hands)	15.5	25.0	1.1	4.3
18	Unable to tell hot (feet)	40.5	38.5	− 0.5	0.5
19	Problem with vomiting	6.0	5.8	2.1	2.3
20	Problem with diarrhea	20.2	30.8	− 1.7	15.3
21	Problem with fainting/dizziness	10.7	19.2	− 8.3	10.6
22	Difficulty bathing	22.6	34.6	− 0.2	17.4
23	Difficulty dressing	21.4	34.6	1.4	22.5
24	Difficulty walking	40.5	59.6	3.9	28.6
25	Difficulty getting on/off toilet	22.6	36.5	2.6	15.8
26	Difficulty using utensils	19.0	30.8	− 1.0	10.1

For the selected Norfolk QOL-DN items, substantial impairment was defined as a response of ‘moderate problem’ or ‘severe problem’

Changes from baseline at week 66 reveal increased percentages of patients in the placebo arm exhibiting problems on Norfolk QOL-DN items, while there are mostly small increases, and even slight decreases, in the percentage of patients in the inotersen arm reporting problems (Table 2). Areas where substantial increases in problems in the placebo arm were accompanied by only slight increases, or even decreases in problems in the inotersen arm included difficulty moving fingers, feeling unsteady on their feet, problems descending stairs, problems with diarrhea, problems with dizziness/fainting, and difficulty bathing, dressing, and walking.

Among the selected SF-36v2 items (Table 3), at week 66 patients receiving placebo more frequently (by at least 15%) reported being ‘limited a lot’ in many aspects of physical functioning, including moderate activities; lifting/carrying groceries; climbing several flights of stairs; bending, kneeling, or stooping; and walking several hundred yards than those receiving inotersen. Patients receiving placebo also more frequently reported quite a bit/extreme disruption of physical and emotional health on social activities at week 66 than did patients in the inotersen arm. Changes from baseline at week 66 showed large increases in percentages of patients in the placebo arm experiencing problems, while there were either slight increases or decreases in the percentage of patients in the inotersen arm reporting problems in most aspects of physical functioning and role limitation due to physical health problems, as well as health interference in social functioning (Table 3).

**Table 3:** Percentage of patients with SF-36v2 item responses indicating substantial impairment at week 66, and change in percentage from baseline to week 66, by treatment arm

Item #	Content	Week 66		Change from baseline to week 66	
		Inotersen (%)	Placebo (%)	Inotersen (%)	Placebo (%)
PF01	Vigorous activities	75.9	82.7	− 5.3	4.7
PF02	Moderate activities	42.5	59.6	− 3.7	22.3
PF03	Lifting/carrying groceries	32.6	51.9	− 2.3	19.7
PF04	Climbing several flights	56.3	75.0	− 2.2	19.1
PF05	Climbing one flight	33.3	46.2	3.1	27.5
PF06	Bending/kneeling/stooping	39.1	63.5	− 1.5	27.9
PF07	Walking more than 1 mile	59.8	65.4	− 1.2	14.5
PF08	Walking several hundred yards	32.2	53.8	− 5.9	21.6
PF09	Walking 100 yards	24.1	32.7	6.2	19.1
PF10	Bathing/dressing	16.1	19.2	7.6	14.1
RP01	Cut down amount of time working	32.2	55.8	− 6.5	25.3
RP02	Accomplished less	44.8	57.7	2.4	18.7
RP03	Limited in kind of work/activity	43.7	59.6	− 2.5	12.2
RP04	Had difficulty performing work/activity	43.7	61.5	− 4.4	17.5
SF01	Health interfered with social activities	11.5	32.7	− 7.6	15.7
BP02	Pain interfered with work/activities	20.7	32.7	− 1.2	3.9

For PF items, substantial impairment was defined as a response of ‘limited a lot’. For RP items, substantial impairment was defined as a response of ‘all of the time’ or ‘most of the time’. For SF and BP items, substantial impairment was defined as a response of ‘extremely’ or ‘quite a bit’

*BP* bodily pain, *PF* physical functioning, *RP* role-physical, *SF* social functioning

## Discussion

We showed that inotersen treatment preserved or improved QOL over 66 weeks in patients with hATTR amyloidosis with polyneuropathy. Several domains on the Norfolk QOL-DN and the SF-36v2 were stable or showed improvements in patients treated with inotersen compared to placebo, for which worsening in mean scores from baseline to week 66 was observed. The effects were more pronounced for physical functioning, role-physical, bodily pain, social functioning, and role-emotional domains. Responder analyses supported these findings. Patients receiving placebo more frequently exhibited clinically meaningful worsening in Norfolk QOL-DN total scores, as well as in domains for ADLs, large fiber/physical functioning, and symptoms, while patients receiving inotersen more often showed either clinically meaningful improvements or no change from baseline in the outcomes. A significantly larger percentage of patients receiving inotersen showed improvements on total scores and the large fiber/physical functioning domain than placebo patients. In addition, a significantly larger percentage of patients receiving inotersen were observed to exhibit clinically meaningful improvement for global measures of both SF-36v2 physical (PCS) and mental (MCS) health outcomes, as well as on domains capturing physical functioning, social functioning, role-emotional, and mental health than for those receiving placebo.

Previous analyses of drug treatment for patients with hATTR amyloidosis, including tafamidis [25–29], diflunisal [21], patisiran [30], and inotersen [13], have shown preserved or positive effects on overall QOL. The current analysis, however, is the first to examine the impact of any treatment for hATTR amyloidosis on specific aspects of functioning and activities of daily living.

The current descriptive examination of responses to items on the Norfolk QOL-DN and the SF-36v2 provides important context for interpreting the impact of inotersen on patients’ daily activities and functioning. Inotersen was more effective than placebo at preserving or improving outcomes related to sleep; to everyday physical functioning, including walking up and down stairs, carrying groceries, walking, and movement of fingers; having less impairment in social functioning and work-related outcomes; and to having less trouble engaging in everyday activities such as bathing, dressing, and handling eating utensils. Responses to items related to neuropathic pain, such as pain that interferes with sleep or bedsheets rubbing against the skin being a noxious stimulus (allodynia), indicate that inotersen treatment, relative to placebo, significantly improved pain, which is consistent with reduced nerve injury resulting in less neuropathic pain. Patients receiving placebo were more likely to show impairment over time for all of these outcomes, while patients receiving inotersen showed improvements or slight impairments in most of these outcomes from baseline to week 66.

One notable finding from the item-level analysis was a larger difference between inotersen and placebo groups on exhibiting substantial limitations at week 66 on the SF-36v2 physical functioning item for walking several hundred yards, yet smaller differences between groups for the items examining walking 100 yards or walking more than a mile. For both of these latter items, slightly more patients in the inotersen group than the placebo group showed substantial difficulties at baseline, which likely led to underestimating the differences at week 66. More revealing is the magnitude of changes from baseline in the percentages of patients having substantial difficulty with walking 100 yards (6% for inotersen vs. 19% for placebo) or with walking more than a mile (− 1% for inotersen (improvement) vs. 15% for placebo (worsening)).

It should also be noted that there was little change in the impact on QOL of small fiber and autonomic neuropathy throughout the study, even for patients in the placebo arm. The mean increase in the Norfolk QOL-DN small fiber and autonomic neuropathy domains from baseline to week 66 was less than one point in both treatment arms. Further, less than one-third of the sample in the placebo arm showed clinically meaningful worsening in these domains from baseline to week 66. Analysis of change in Norfolk QOL-DN small fiber and autonomic neuropathy scores in the placebo arm of the phase 3 APOLLO study of patients with hATTR amyloidosis showed an increase of 2.8 and 0.8 points, respectively, over 18 months, so also showing minimal change [31]. Thus, it may be that the majority of patients in the NEURO-TTR trial had already reached a critical progression in small fiber and autonomic neuropathy, which are early manifestations of the disease, producing a ceiling effect. At the same time, it should be noted that we did see improvement in autonomic neuropathy for patients receiving inotersen relative to placebo: the proportion of patients in the inotersen arm experiencing autonomic symptoms of fainting/dizziness and diarrhea decreased from baseline to week 66 (− 8.3% and − 1.7%, respectively), while the proportion of patients in the placebo arm experiencing these symptoms increased from baseline to week 66 (10.6% and 15.3%, respectively). Another possibility is that the Norfolk QOL-DN small fiber and autonomic neuropathy domains scores lack the sensitivity needed to detect actual changes in this outcome.

There are limitations of the current study that should be considered. One limitation is the lack of end-stage patients in the NEURO-TTR trial. By the study protocol, only patients in Stage 1 or Stage 2 severity were eligible for enrollment; patients in Stage 3 (not ambulatory) were excluded. It is possible that the impact of inotersen might be different for patients with highly advanced disease than for those in the earlier stages, although it should be noted that many aspects of functioning (e.g., walking, climbing stairs) would not be measurable in non-ambulatory patients. Another limitation was the duration of the treatment period, which was 66 weeks. Patients in either treatment arm who completed the trial were eligible to enroll in an open-label extension (OLE) of the trial in which they would receive 300 mg inotersen once weekly for up to 5 years. Future results from the OLE study will address the long-term impact of inotersen on patients’ QOL. The responder-level MIC values for SF-36 domain and summary scores that were used in the responder analyses were established based on a US general population sample, and have not been tested in an hATTR amyloidosis sample, where the magnitude of differences in scores over time corresponding to meaningful change may differ. Also, the designation of item responses characterized as indicating substantial impairment or not substantial impairment was based on arbitrary, subjective judgment; response choices could be dichotomized differently. Finally, we note that all analyses conducted here were post hoc and exploratory; they were performed to better understand the impact of inotersen on more granular components of QOL, since the pre-specified endpoints of the NEURO-TTR study were based on total and summary scores of QOL measures.

In conclusion, patients with hATTR amyloidosis with polyneuropathy treated with inotersen showed better preservation or improvements of QOL at 66 weeks than did patients receiving placebo, particularly with respect to physical functioning, activities of daily living, neuropathic symptoms, role limitations due to physical and emotional functioning, and social functioning.
